# New Appliances and Things Medical

**Published:** 1899-02-04

**Authors:** 


### NEW APPLIANCES AND THINGS MEDICAL.

[We shall be glad to receive, at our Office, 28 & 29, Southampton Street, Strand, London, W.C., from the manufacturers, specimens of all new preparations and appliances which may be brought out from time to time.]

### CHLOROBROM.

(Burgoyne, Burbidges, and Co., 12 and 16, Coleman Street, London, E.C.)

Though highly appreciated as a hypnotic by certain physicians familiar with its application, chlorobrom is not as generally known as its merits deserve. As prepared by Messrs. Burgoyne and Burbidges it contains 30 grains of chloralamid to the ounce, a dose which is sufficient in ordinary cases to induce a refreshing sleep of some hours' duration. It will be found of special use in cases of hysteria, nerve overstrain, and mental excitement, and, as far as is known, its use is attended with no dangerous or unpleasant after effects.

### BREAKFAST HEALTH BEVERAGES.

(Breakfast Health Beverage Company, Redditch.)

This new breakfast beverage is offered to the public in the hope that it may supply a substitute or an alternative for the usual cup of tea or coffee at this meal. It has the advantage of being readily made, namely, by preparing as ordinary coffee. It is pleasant in flavour, something like that of modified cocoa, and for those who seek variety and novelty it may afford a welcome change from the more usual beverages. It is stated to be solely prepared from celery and parsnips. It appears to consist for the main part of starch, cellulose, sugar, and dextrin, with certain aromatic bodies, which impart the characteristic flavour.

### D.C.L. EXTRACT OF MALT AND COD LIVER OIL.

(The Distillers Company, Limited.)

The combination of the D.C.L extract of malt with cod liver oil should prove of considerable value to those suffering from defective nutrition, constituting in itself at once a food and an adjuvant to digestion. The cod liver oil is of excellent quality, and the malt extract with which it is combined is of the same high standard of excellence as that which is familiarly known as the D.C.L. brand.

### "JOHANNIS" NATURAL MINERAL WATER LITHIATED.

(The Apollinaris Company, Limited, 4, Stratford Place, Oxford Street, London, W.)

No doubt everything has its uses, and among them strongly mineralized mineral waters. It must be confessed, however, that Dame Nature does not always provide these waters in exactly the form and constitution which is likely to be most useful. Lithium, for example, certainly exists in natural waters, but while with some of them one has to drink a great deal of water for a very little lithium, in others, while we succeed in getting a fair quantity of the drug we want, we have to take with it a good many other things which we don't desire at all. Hence it has happened that lithium has usually been given in the form of an artificially aërated water. Unfortunately the fact that lithium was introduced as a medicine led to lithia water being ordered and made of medicinal strength. A great many people, however, find some benefit from using this drug in very weak doses, and to meet their wants the Apollinaris Company have arranged to supply a lithiated Johannis water, each small bottle of which contains one grain of lithium bicarbonate. Those, then, who like these small doses, that is those who find that they derive more benefit from the constant dietetic use of lithium than from its occasional employment as a medicine, and there are many who do so, are thus able to combine the advantages of a natural table water with the small dose of lithium which they require.

